# Thermal Energy Storage Using Phase Change Materials in High-Temperature Industrial Applications: Multi-Criteria Selection of the Adequate Material

**DOI:** 10.3390/ma17081878

**Published:** 2024-04-18

**Authors:** Luisa F. Cabeza, Franklin R. Martínez, Emiliano Borri, Svetlana Ushak, Cristina Prieto

**Affiliations:** 1 GREiA Research Group, Universitat de Lleida, Pere de Cabrera s/n, 25001 Lleida, Spain; rodrigo.martinez@udl.cat (F.R.M.); emiliano.borri@udl.cat (E.B.); 2 Center for Advanced Study of Lithium and Industrial Minerals (CELiMIN), University of Antofagasta, Campus Coloso, Av. Universidad de Antofagasta, Antofagasta 02800, Chile; svetlana.ushak@uantof.cl; 3 University of Seville, Department of Energy Engineering, Camino de los Descubrimientos s/n, 41092 Seville, Spain; cprieto@us.es

**Keywords:** thermal energy storage, phase change materials, multi-criteria selection of materials

## Abstract

Thermal energy storage (TES) plays an important role in industrial applications with intermittent generation of thermal energy. In particular, the implementation of latent heat thermal energy storage (LHTES) technology in industrial thermal processes has shown promising results, significantly reducing sensible heat losses. However, in order to implement this technology, a proper selection of materials is important. In this study, a new multi-criteria phase change material (PCM) selection methodology is presented, which considers relevant factors from an application and material handling point of view, such as hygroscopicity, metal compatibility (corrosion), level hazard, cost, and thermal and atmospheric stability. The methodology starts after setting up the system requirements where the PCM will be used, then a material screening is able to find all possible candidates that are listed with all available properties as listed before. Then, a color map is produced, with a qualitative assessment of material properties drawbacks, hazard level, melting enthalpy, and price. The experimentation starts with a preliminary set of tests on hygroscopicity and one-week corrosion test, which allows disregarding PCMs and selecting a short list of potential PCMs that would need further characterization before the final selection.

## 1. Introduction

Thermal energy storage (TES) is a key component in the optimization of industrial processes, in applications with intermittent thermal energy generation, such as solar thermal systems or waste heat recovery, for which a suitable thermal storage system is essential [1]. TES systems have been developed as useful engineering solutions to reduce the gap between energy supply and demand in cooling or heating applications by storing extra energy generated during peak collection hours and dispatching it during off-peak hours [2,3]. Large-scale applications such as power plants, geothermal power units, nuclear power plants, smart textiles, buildings, the food industry and solar energy capture and storage are ideal candidates for TES systems [4]. Latent thermal energy storage is an attractive technology for industry when integrated into thermal processes, reducing potentially sensible heat losses in the heating and cooling processes needed to reach optimal temperatures, and allowing heat to be stored between cycles. However, to implement this technology it is necessary to select the appropriate materials within the required working temperature ranges for each application [5].

The literature shows that there are many materials that can be used as phase change materials (PCMs) [6,7], but researchers and practitioners still struggle to choose the right PCM for each application. The literature shows that a large number of the selection methodologies of suitable PCMs adopted by many authors [8,9,10,11,12] in different studies consider a limited number of factors as relevant for the selection. Traditionally these factors are melting enthalpy, melting temperature, and the amount of energy that can be stored and released. However, from an application and material handling point of view, other factors are also crucial and important to be considered in the PCM selection methodology, such as hygroscopicity, metal compatibility (corrosion), level hazard, cost, and thermal and atmospheric stability. In addition, during the process of identifying potential PCMs, it is common to obtain a large number of potential materials with properties and characteristics compatible with the final application. Nevertheless, carrying out the full characterization for all these potential candidates involves significant economic investment, time, effort, and dedication. In this context, the need for a methodology that allows, during the initial stages, for the reduction in the number of potential materials to a smaller group of real candidates for which it is worthy to perform full characterization and exhaustive analyses for the final selection arises.

**Figure 1:**
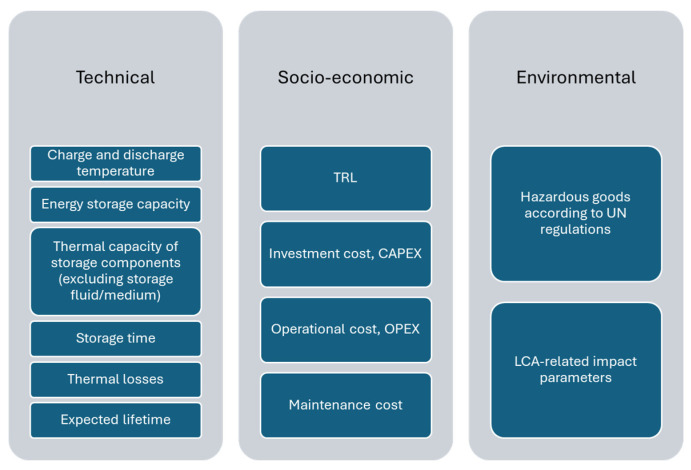
Classification of KPIs to evaluate TES systems (adapted from [13]).

Selection of PCM candidates does not depend only on the melting temperature, the temperature at which the energy will be released, which depends on the application requirements, but also on many other properties and parameters. Palomba and Frazzica [13] divided the key performance indicators (KPIs) identified for TES in three groups, technical, socio-economic, and environmental (Figure 1). In fact, already in 2016, Miró et al. [14] included health hazard and cycling and thermal stability as key parameters when selecting a suitable PCM. Cycling and thermal stability are usually included in PCM selection, while health hazard is still not included in the scientific literature as a KPI. The form, condition, and inherent properties of a material determines its health hazard, which is usually found in the manufacturer information, such as material safety data sheets. This parameter is especially important in high temperature applications, since the degree of personal protective equipment required to work safely with the material usually increases with temperature.

Moreover, the selection methodology should be adapted to each case by selecting the right KPIs and more importantly, the range selected for each KPI. For example, Gasia et al. [15] adapted the previous methodology developed by Miró et al. to a partial load evaluation of PCM tanks working at a 120–200 °C temperature range, and this was achieved by extending the cycling stability evaluation of the PCM to one hundred cycles and cycling them under an atmosphere which simulates the boundary conditions of the further pilot plant experimental setup testing and using a higher sample mass.

Maldonado et al. [16] used the same methodology to select a PCM to be used for TES storage in a solar system using Fresnel collectors, with a required melting temperature between 210 °C and 270 °C. An important outcome of this paper is that it states that the results found experimentally do not always agree with those found in the literature, especially data on melting temperatures and melting enthalpies. Therefore, other authors continue working on benchmarking PCMs for different applications. For example, Navarro et al. [17] experimentally characterized different PCMs to be used in building applications, where the main change was the extension of the thermal stability analysis to 10,000 cycles, equivalent to 30 years of buildings use, and analyzing also the thermal conductivity of the PCMs.

In 2020, Zsembinszki et al. [18] went a step further in the application of the selection methodology including a step-based method. This change was due to the high amount of potential PCMs found in the given application, a climatization system for residential buildings which includes an active PCM tank. The methodology was based on three steps. The first step was a thorough review of potential PCM candidates available in the scientific literature and available commercially. The second step excluded PCMs that were not suitable either because of health hazard issues, compatibility with the storage tank container (corrosion), or thermophysical properties below the required targets. Finally, the third step consisted of the development of a decision matrix as a tool to make a final selection based on objective criteria. This decision matrix was built following parameters that were considered as key for the given application (Table 1). Moreover, the authors considered different scenarios for the weighing of each criterion. It should be highlighted that this paper includes criteria such as availability and price to select the right PCM.

**Figure 2:**
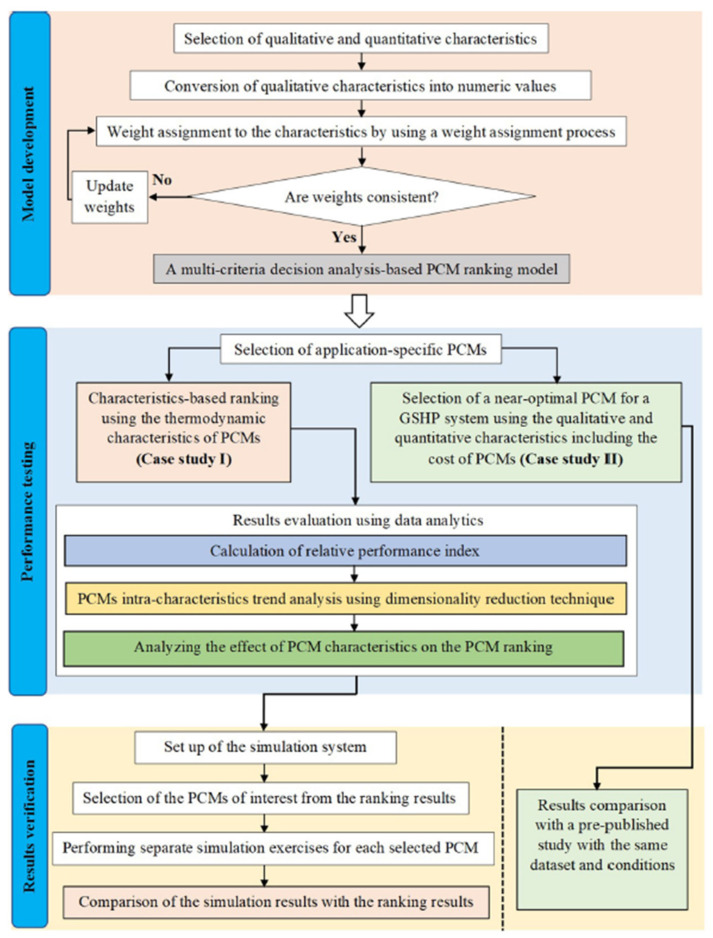
Outline of the selection strategy used by Awan et al. [19].

This last methodology can be considered a multi-criteria decision-making analysis (MCDA), which was recently also applied by Awan et al. [19] (Figure 2). The effectiveness of this strategy was tested in two case studies. In the first case study, a sample dataset of PCMs was used to rank PCMs for building applications based on the four major thermodynamic properties of the PCMs; in the second case study, several qualitative and quantitative characteristics of PCMs including cost were used to select a near-optimal PCM from a list of eight PCMs for a thermal energy storage system integrated with a ground source heat pump system. Although the method proved to be effective, the authors stated that its main drawback is its subjectivity.

Similarly, Akgün et al. [20] used a MCDA to select carbon-based nanomaterials as PCM additive. Seven evaluation criteria were determined (melting point temperature change, latent heat change, thermal conductivity enhancement, leakage, greenhouse gas, cost, and agglomeration) based on the literature information and the best additive was selected.

Based on Refs. [8,9,10,11,12], given the need for better methods of selecting suitable PCMs, this paper presents a novel multi-criteria PCM selection methodology, aimed primarily at high-temperature industrial applications. In contrast to traditional PCM selection methodologies, the methodology presented in this paper considers a more comprehensive point of view, considering crucial factors related to application and handling such as hygroscopicity, hazard level, corrosion resistance, cost, and thermal and atmospheric stability and. In addition, another one of the advantages of this methodology is the identification of, within the first stage, candidates which are not worthy of full characterization.

In this study, this methodology is applied to high-temperature PCM selection for industrial applications. In this case, study, the system requirements were as follows:

- Temperature range of operation: 400 °C to 600 °C.
- High latent heat storage capacity: high phase change enthalpy.
- Easiness in handling and not imposing health hazard.
- Thermal cycling stability: thermal properties need to remain almost constant during a certain number of thermal cycles.
- Thermal stability: maximum working temperature of at least 50 °C over the range of operation.
- Compatibility with the metal selected for the metal wool and the storage container that will contain the PCM.
- Suitable price.
- Atmospheric stability: PCM should be stable in the atmospheric conditions of the storage container.

## 2. Methodology

### 2.1. Selection Methodology

**Figure 3:**
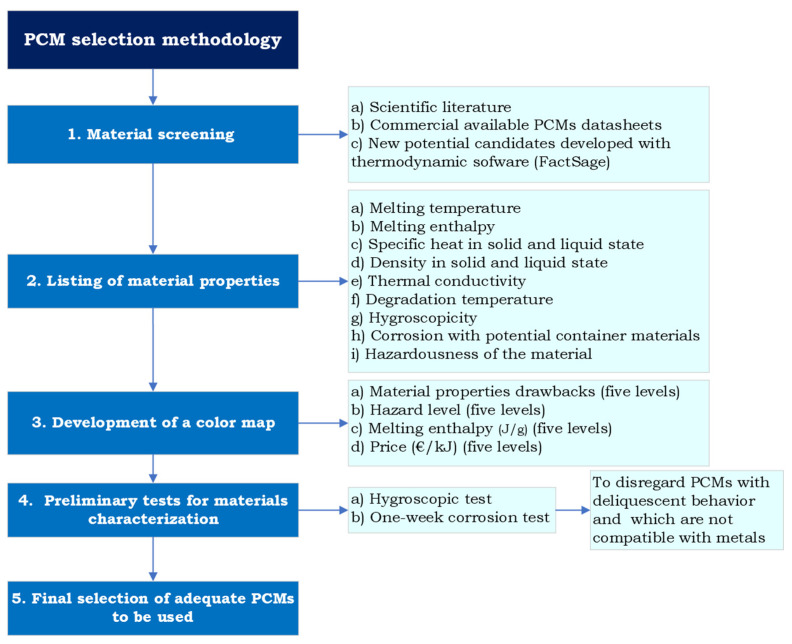
PCM selection methodology.

**Figure 4:**
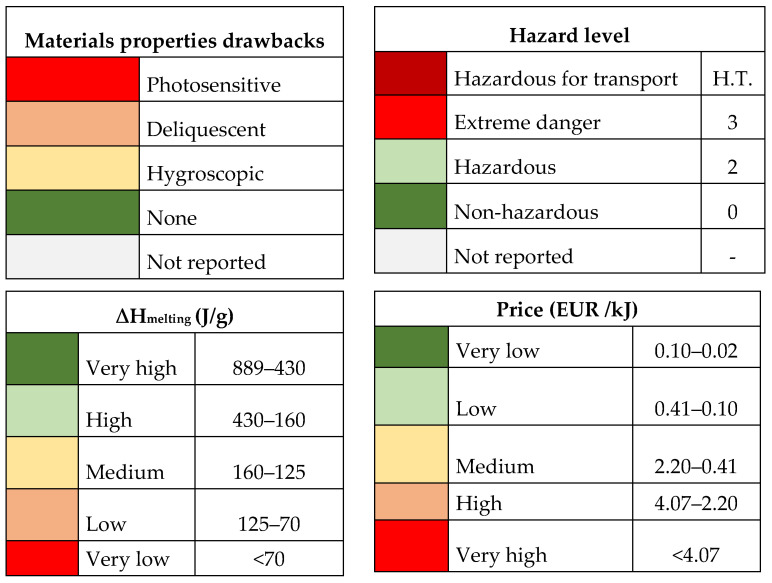
Color map developed for several parameters to highlight suitability of the potential materials.

The methodology developed to perform the material selections had the following steps (Figure 3):

1. Materials screening
  The screening was three-fold. First, the scientific literature was thoughtfully scanned to find identified candidates and their disclosed properties. Then, commercially available PCMs were listed. The companies considered were Rubitherm (Germany), PCM Products (United Kingdom), and PLUSS (India). And third, the software FactSage Education 8.3 was used to find potential new candidates.
2. Listing of materials’ properties
  When reported, different properties were listed. The properties were collected from the literature from the materials’ data sheets. The considered properties were melting temperature, melting enthalpy, specific heat in solid and liquid state, density in solid and liquid state, thermal conductivity, degradation temperature, hygroscopicity, corrosion with potential container materials, and hazardousness of the material (following the standard NFPA 704 [21]).
3. Development of a color map
  To facilitate the selection, a color map was developed. For the different key parameters, acceptable and non-acceptable levels were defined, and a color classification was developed. Some examples of such parameters and levels are shown in Figure 4.
4. Preliminary tests for materials characterization
  To disregard materials that did not comply with the requirements settled, two tests were carried out. First, a hygroscopic analysis was applied to potential PCMs, such as inorganic salts, identified as hygroscopic or deliquescent in their data sheets. If a salt was pointed out as not adequate, all PCMs with that salt would be disregarded. Second, a one-week corrosion test was performed with the identified PCMs with the pre-selected metals to be used in contact with the PCM.
5. Final selection of adequate PCMs to be used.

### 2.2. Analytical Methods

The hygroscopic analysis was carried out leaving a known quantity of salt (5 g), not the PCM itself, but the salts that would be mixed to become the PCM, in a watch glass during 8 h at atmospheric conditions and weighing the samples every hour. The test was carried out with the salt as it is and also after drying it in an oven at 200 °C for 12 h. The first test would be similar to the real conditions, and the second would allow to better measurement of water uptake. Moreover, both samples were dried again after the test to see if they would look as before the water uptake.

**Figure 5:**
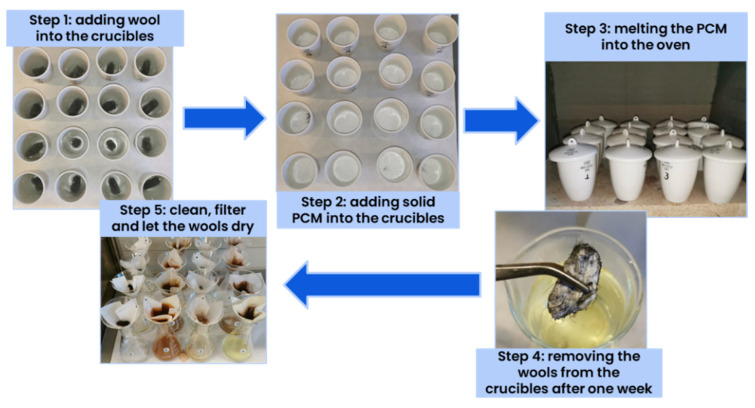
Corrosion tests methodology.

The corrosion test was carried out by immersing the selected metal in the molten PCM following the tests shown in [22]. The temperature at which the oven was set up depended on the melting temperature of the PCM. In this selection criterion, the corrosion tests were performed only for 1 week to use it as a selection tool. The selected PCMs would go to a 3-month test later to have the data to ensure good metal-PCM compatibility. In this study, the tests were performed with stainless steel (314 and 434) and with Alloy 20 metal wool. At least 2 samples of each pair were tested to ensure repeatability, following the methodology shown in Figure 5.

**Figure 6:**
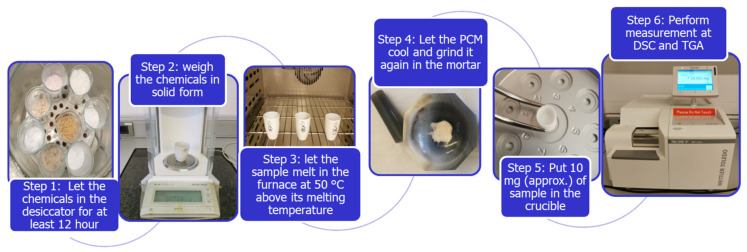
Sample preparation for DSC and TGA/DSC measurements.

The thermophysical evaluation was carried out by differential scanning calorimetry (DSC) and thermogravimetric analysis (TGA). The phase change temperature, enthalpy, and thermal behavior analysis of the selected PCMs were determined by DSC using a STARe SYSTEM DSC 3+ from METTLER TOLEDO. The accuracy of the equipment is ±0.1 °C for temperature results and ±3 J/g for enthalpy results. The decomposition temperature, melting enthalpy and melting temperature, and thermal behavior were studied by TGA/DSC. The equipment used to carry out this analysis is the STARe SYSTEM TGA/DSC 3+ from METTLER TOLEDO (Barcelona, Spain). This equipment has a balance with a precision of ±0.00001 g which allows the loss of mass associated with the decomposition process to be quantified. The accuracy of the equipment is ±0.1 °C for temperature results and ±3 J/g for enthalpy results. In both techniques, a small amount of sample (around 10 mg) was placed in sapphire crucibles (70 µL), closed in DSC and open in TGA/DSC, and the tests were carried out in an inert N_2_ atmosphere. The samples preparation for both techniques is shown in Figure 6.

The measurement conditions for DSC were a temperature range between 50 °C above and below the melting point of the PCM, a heating/cooling rate of 1 K/min, and 3 cycles. On the other hand, the measurement conditions for TGA/DSC were a temperature range from 25 °C to 1000 °C and a heating/cooling rate of 1 K/min.

## 3. Results

After the first step of the selection methodology, a total of 27 materials were found with potential to be selected. The materials screening is listed in Table 2.

After completing Table 2 with the maximum information found in the literature and in the materials data sheets, a color map was developed to help disregard non-acceptable materials (Table 3). In this step, several decisions have to be made, which are directly related to the requirements of the application and with educated knowledge from the personnel carrying out such selection. For example (Figure 1):

1. Melting enthalpy: This property is the one that is directly related to the final energy density of the storage system, therefore the higher the melting enthalpy the better. Keeping this in mind, in this case, materials with melting enthalpies between 889 and 430 J/g were considered very high (dark green), materials with melting enthalpies between 430 and 160 J/g were considered high (light green), materials with melting enthalpies between 160 and 125 J/g were considered medium (yellow), materials with melting enthalpies between 125 and 70 J/g were considered low (orange), and materials with melting enthalpies lower than 70 J/g were considered very low (red).
2. Price: Although the price of the storage media is only a part of the total cost of the system, it is clear that the lower the price of the PCM the better. It should be highlighted that the price considered here was not the cost per kg of material, which is the usual purchase cost, but the cost per energy unit, so the comparison between materials would be fairer. Here also five levels were defined, with PCMs with a price higher than 4.07 EUR /kJ were considered very high (red), PCMs with a price between 4.07 and 2.20 EUR /kJ were considered high (orange), PCMs with a price between 2.20 and 0.41 EUR /kJ were considered medium (yellow), PCMs with a price between 0.41 and 0.10 EUR /kJ were considered low (light green), and those with a cost between 0.10 and 0.02 EUR /kJ were considered very low (dark green)
3. Hazard level: The standard NFPA 704 was used to label the hazard level of the materials. Here four levels were identified, from non-hazardous (dark green), hazardous (light green), extreme danger (red), and hazardous for transport (dark red).
4. Within the material properties, in the first stage the hygroscopicity, deliquescence, and photosensitivity were identified as properties that would jeopardize the materials handling, especially during charging of the storage tanks. Therefore, this information was listed for all materials. Here, five levels were considered, marking photosensitive materials (red), deliquescence materials (orange), hygroscopic materials (yellow), materials where none of these properties would appear (green) or were not reported (grey). This category was not used to discharge materials at this stage, but for further testing in the next step of the methodology.

**Figure 7:**
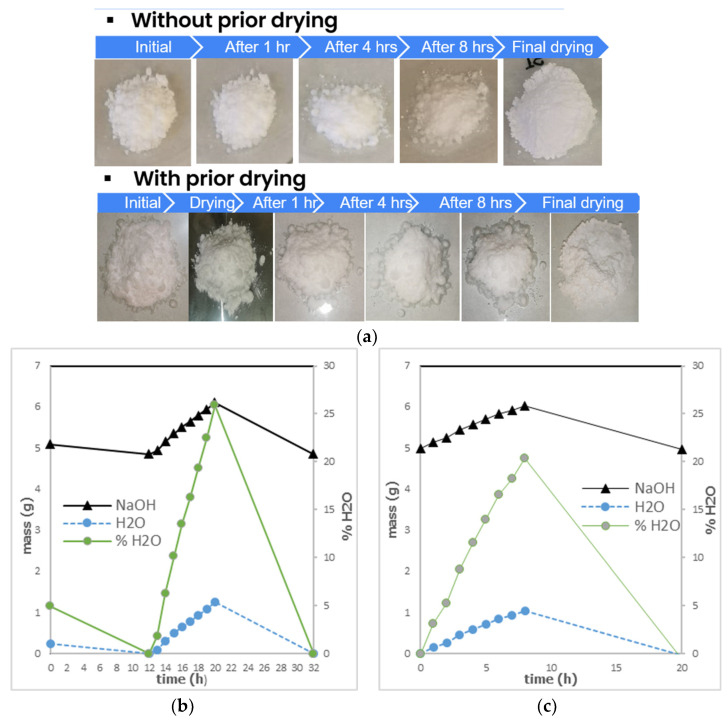
NaOH hygroscopic tests: (**a**) visual inspection of the samples, (**b**) hygroscopic behavior (amount of water absorbed) with prior drying, and (**c**) hygroscopic behavior (amount of water absorbed) without prior drying.

The preliminary hygroscopic test was carried out for ten anhydrous inorganic compounds (compounds required for the preparation of the potential PCMs), under standard environmental conditions (19 °C, 70% RH), which were initially reported as deliquescent in their data sheets. Figure 7 shows an example of a PCM, NaOH in this case, during the hygroscopic test. At the beginning NaOH has a dry appearance. However, after one hour of being exposed to the environment, the appearance of humidity can be noticed, and the first liquid drops appear. After four hours, the appearance of humidity in NaOH is more evident. Furthermore, after eight hours, it can observe clearly that NaOH looks completely wet. Finally, after the drying process, NaOH increased its volume, but its appearance was similar to the initial one. 

Results were translated to mass change, as shown in Figure 7b,c. Materials behave differently with prior drying than without prior drying, absorbing a higher percentage of water after 8 h of exposure to the environment. NaOH absorbed 25.88% of humidity (with prior drying) in 8 h (1.26 g H_2_O), and 20.37% of humidity (without prior drying) in 8 h (1.05 g H_2_O).

**Figure 8:**
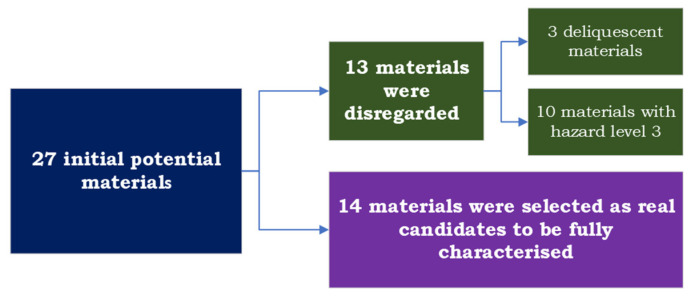
Disregarding materials procedure.

Once the hygroscopicity tests were concluded, those PCMs containing these inorganic compounds with deliquescent behavior were no longer considered for the next stage of preliminary experimental tests. In addition, the materials with hazard level 3 were also no longer considered for the next stage of preliminary experimental tests. This procedure is summarized in Figure 8.

Consequently, fourteen of the initial twenty-seven potential PCMs were considered for corrosion testing and thermophysical evaluation of their properties (TGA/DSC analysis). An example of the results of the one-week corrosion test of one of the PCMs are shown in Table 4. In the case of NaOH, the stainless-steel fibers after one week of being immersed in this PCM were completely disintegrated, while the Alloy 20 fibers retained their integrity. Therefore, the three-month test will be carried out with Alloy 20 metal fiber. 

**Figure 9:**
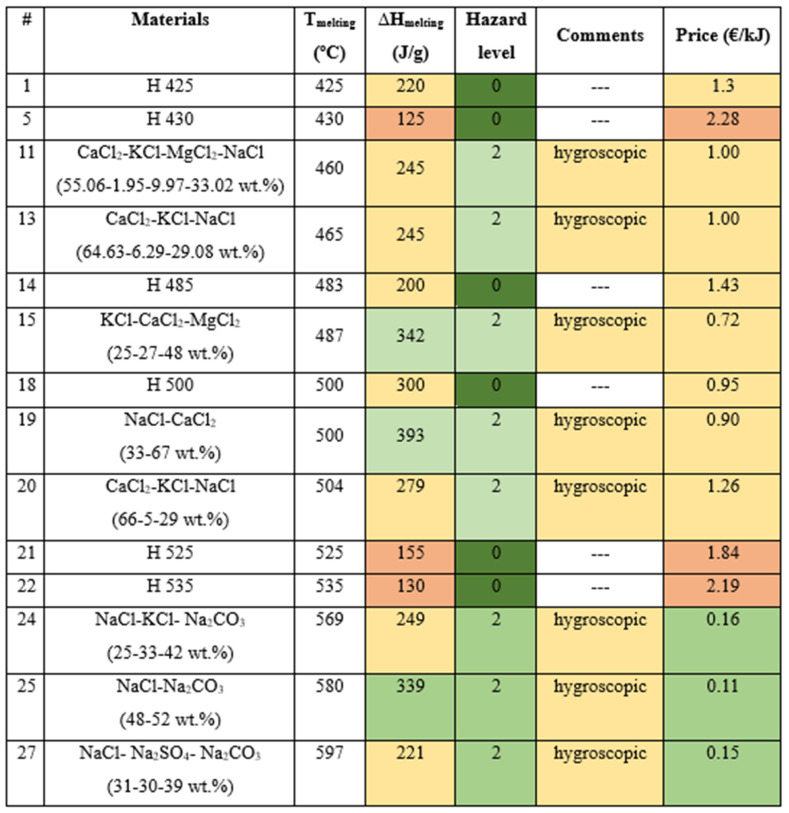
Final potential PCM to be used in the temperature range 400 °C–600 °C. Colors in the figure reflect the compliance of each material with the set requirements (i.e., dark green means high compliance and orange means no compliance).

With all these tests, the preselected PCMs that comply with the requirements set are shown in Figure 9. For final use, a complete characterization is needed, but this expensive characterization would be done only in the PCMs that are real candidates to be used in the application. 

## 4. Conclusions

Based on the initial screening of the literature, in the temperature range of 400 °C to 600 °C, we have identified and compiled the characteristics of a total of 27 potential PCM candidates. However, the application of new criteria added in this work subsequently led to a final selection of 14 potential materials as viable PCMs for more complete characterization and analysis. Using a color map (Figure 9), it can be established that the NaCl-Na_2_CO_3_ mixture (48–52 wt.%) could have high potential as PCM, but its handling in an atmosphere with low humidity should be considered.

In conclusion, we present a novel PCM selection methodology based on multiple criteria specifically designed for high-temperature industrial applications. This methodology comprehensively considers crucial factors related to material implementation and management, including hygroscopicity, hazard level, and corrosion resistance. Through a rigorous selection process involving literature review, commercial availability assessment, and thermophysical property analysis, we identified a total of 27 potential PCM candidates. The new criteria added in this work subsequently led to a final selection of 14 potential materials as viable PCMs for further comprehensive characterization and analysis.

These findings underscore the critical importance of meticulous material selection in driving the implementation of latent heat thermal energy storage (LHTES) technology in industrial thermal processes. By enabling informed decision-making, our methodology significantly contributes to the advancement of high-temperature thermal energy storage technology. This progress, in turn, paves the way for the development of more efficient and sustainable industrial processes, aligning with the growing need for environmentally friendly energy solutions across various industrial sectors. In the future, further research and validation efforts will be essential to optimize the performance and applicability of the PCM candidates identified in real industrial environments.

## Figures and Tables

**Table 1:** Scoring criteria applied by Zsembinszki et al. [18].

Temperature Range [°C]		Enthalpy [kJ/kg]		Availability		Price [EUR /kg]		Maximum Working Temperature [°C]	
Criteria	Value for Decision	Criteria	Value for Decision	Criteria	Value for Decision	Criteria	Value for Decision	Criteria	Value for Decision
T < 2	3	h > 250	3	Yes	3	P < 2.5	3	T_max_ > 120	3
2 < T < 3	2	200 < h < 250	2	No	0	2.5 < P < 5	2	T_max_ < 120 or n.a.	0
3 < T < 4	1	150 < h < 200	1	---	---	5 < P < 10	1	---	---
T > 4 or n.a.	0	h < 150 or n.a.	0	---	---	P > 10 or n.a.	0	---	---

n.a.—not available.

**Table 2:** Potential materials to be used as a PCM in the range of 400 °C to 600 °C.

#	Materials	T_melting_ (°C)	∆H_melting_ (J/g)	Cp _solid_ (J/g·K)	Cp _liquid_ (J/g·K)	ρ _solid_ (kg/m^3^)	ρ _liquid_ (kg/m^3^)	*k* _liquid_ (W/m·K)	Corrosion (mm/Year)	T_degradation_ (°C)	NFPA 704	Ref.
1	H 425	425 [23]	220 [23]	1.54 [23]	-	2100 [23]	-	0.57	-	1400 [23]	N.H.	[23]
2	MnCl_2_-NaCl (64.33-35.67 wt.%)	426 [24]	230 [24]	-	-	-	-	-	-	-	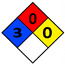	[24]
3	LiF-LiOH (20-80 mol%)	427 [25]	869 [25]	0.80 [25]	1.00 [25]	1600 [25]	-	-	-	-	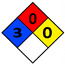	[25]
4	LiF-LiOH (21.33-78.67 wt.%)	431 [24]	889 [24]	-	-	-	-	-	-	-	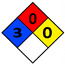	[24]
5	H 430	430 [23]	125 [23]	1.54 [23]	-	2160 [23]	-	0.57 [23]	-	1400 [23]	N.H.	[23]
6	MgCl_2_-KCl (39-61 wt.%)	435 [26]	351 [26]	0.80 [26]	0.96 [26]	2110 [26]	-	0.81 [26]	1.00 [27]	700 [28]	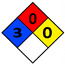	[26,27,28]
7	MgCl_2_-RbCl (21.65-78.35 wt.%)	446 [24]	135.7 [24]	-	-	--	-	-	-	-	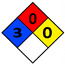	[24]
8	LiCl-MgCl_2_ (49.65-50.35 wt.%)	447 [24]	401 [24]	-	-	--	-	-	-	-	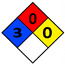	[24]
9	LiF-LiBr (8.62-91.38 wt.%)	450 [24]	275 [24]	-	-	-	-	-	-	-	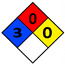	[24]
10	NaCl-MgCl_2_ (48-52 wt.%)	450 [25]	430 [25]	0.92 [25]	1.00 [25]	2230 [25]	-	0.95 [25]	-		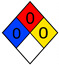	[25]
11	CaCl_2_-KCl-MgCl_2_-NaCl (55.06-1.95-9.97-33.02 wt.%)	460 [25]	245 [25]	-	-	-	-	-	-	--	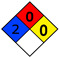	[25]
12	LiOH	462 [29]	873 [29]	-	-	1460 [29]	-	-	-	-	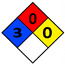	[29]
13	CaCl_2_-KCl-NaCl (64.63-6.29-29.08 wt.%)	465 [25]	245 [25]	-	-	-	-	-	-	-	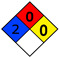	[25]
14	H 485	483 [23]	200 [23]	1.55 [23]	-	2220 [23]	-	0.57 [23]	-	800 [23]	N.H.	[23]
15	KCl-CaCl_2_-MgCl_2_ (25-27-48 wt.%)	487 [25]	342 [25]	0.80 [25]	-	2530 [25]	-	0.88 [29]	-	-	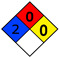	[25,29]
16	Li_2_CO_3_-K_2_CO_3_ (47-53 wt.%)	488 [25]	342 [25]	1.03 [25]	1.34 [25]	2220 [25]	-	1.99 [25]	-	530 [30]	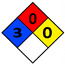	[25]
17	Li_2_CO_3_-Na_2_CO_3_ (44-56 wt.%)	496 [25]	370 [25]	1.80 [25]	2.09 [25]	2320 [25]	-	2.09 [25]	-	530 [30]	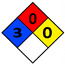	[25]
18	H 500	500 [23]	300 [23]	1.55 [23]	-	2200 [23]	-	0.57 [23]	-	800 [23]	N.H.	[23]
19	NaCl-CaCl_2_ (33-67 wt.%)	500 [25]	393 [25]	0.84 [25]	1.00 [29]	2160 [25]	-	1.20 [25]	-	-	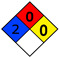	[25,29]
20	CaCl_2_-KCl-NaCl (66-5-29 wt.%)	504 [29]	279 [29]	1.17 [29]	1.00 [29]	2150 [29]	-	1.00 [29]	-	-	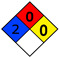	[29]
21	H 525	525 [23]	155 [23]	1.56 [23]	-	2350 [23]	-	0.57 [23]	-	1000 [23]	N.H.	[23]
22	H 535	535 [23]	130 [23]	1.57 [23]	-	2320 [23]	-	0.56 [23]	-	1000 [23]	N.H.	[23]
23	Ca(NO_3_)_2_	560 [29]	145 [29]	-	-	2113 [29]	-	-	-	500 [31]	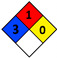	[29]
24	NaCl-KCl-Na_2_CO_3_ (25-33-42 wt.%)	569 [7]	249 [7]	1.34 [7]	1.41 [7]	1700 [7]	2000 [7]	0.50 [7]	-		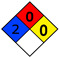	[7]
25	NaCl-Na_2_CO_3_ (48-52 wt.%)	580 [7]	339 [7]	1.3 [7]	-	2000 [7]	-	0.60 [7]	-		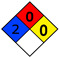	[7]
26	Li_2_CO_3_-Na_2_CO_3_-K_2_CO_3_ (22-16-62 wt.%)	580 [25]	288 [25]	1.80 [25]	2.90 [25]	2340 [25]	-	1.95 [25]	-	827 [30]	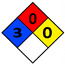	[25]
27	NaCl-Na_2_SO_4_-Na_2_CO_3_ (31-30-39 wt.%)	597 [7]	221 [7]	1.20 [7]	1.30 [7]	2000 [7]	-	0.50 [7]	-		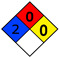	[7]

N.H.—non-hazardous.

**Table 3:** Classification of the main PCMs properties by levels.

#	Materials	T_melting_ (°C)	∆H_melting_ (J/g)	Hazard Level	Comments	Price (EUR /kJ)
1	H 425	425	220	0	--	1.30
2	MnCl_2_-NaCl (64.33-35.67 wt.%)	426	230	3	photosensitive	0.24
3	LiF-LiOH (20-80 mol%)	427	869	3	hygroscopic	352.12
4	LiF-LiOH (21.33-78.67 wt.%)	431	889	3	hygroscopic	0.35
5	H 430	430	125	0	--	2.28
6	MgCl_2_-KCl (39-61 wt.%)	435	351	3	hygroscopic	0.28
7	MgCl_2_-RbCl (21.65-78.35 wt.%)	446	136	3	hygroscopic	--
8	LiCl-MgCl_2_ (49.65-50.35 wt.%)	447	401	3	hygroscopic	--
9	LiF-LiBr (8.62-91.38 wt.%)	450	275	3	deliquescent	--
10	NaCl-MgCl_2_ (48-52 wt.%)	450	430	3	hygroscopic	0.25
11	CaCl_2_-KCl-MgCl_2_-NaCl (55.06-1.95-9.97-33.02 wt.%)	460	245	2	hygroscopic	1.00
12	LiOH	462	873	3	deliquescent	0.38
13	CaCl_2_-KCl-NaCl (64.63-6.29-29.08 wt.%)	465	245	2	hygroscopic	1.00
14	H 485	483	200	0	--	1.43
15	KCl-CaCl_2_-MgCl_2_ (25-27-48 wt.%)	487	342	2	hygroscopic	0.72
16	Li_2_CO_3_-K_2_CO_3_ (47-53 wt.%)	488	342	3	hygroscopic	0.99
17	Li_2_CO_3_-Na_2_CO_3_ (44-56 wt.%)	496	370	3	hygroscopic	0.21
18	H 500	500	300	0	--	0.95
19	NaCl-CaCl_2_ (33-67 wt.%)	500	393	2	hygroscopic	0.90
20	CaCl_2_-KCl-NaCl (66-5-29 wt.%)	504	279	2	hygroscopic	1.26
21	H 525	525	155	0	--	1.84
22	H 535	535	130	0	--	2.19
23	Ca(NO_3_)_2_	560	145	3	deliquescent	0.12
24	NaCl-KCl-Na_2_CO_3_ (25-33-42 wt.%)	569	249	2	hygroscopic	0.16
25	NaCl-Na_2_CO_3_ (48-52 wt.%)	580	339	2	hygroscopic	0.11
26	Li_2_CO_3_-Na_2_CO_3_-K_2_CO_3_ (22-16-62 wt.%)	580	288	3	hygroscopic	0.22
27	NaCl-Na_2_SO_4_-Na_2_CO_3_ (31-30-39 wt.%)	597	221	2	hygroscopic	0.15

**Table 4:** Results of the corrosion tests.

PCM	Stainless Steel 314		Stainless Steel 434		Alloy 20	
Before tests	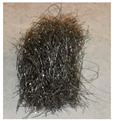	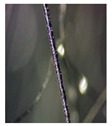	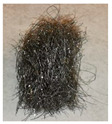	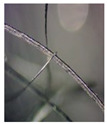	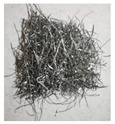	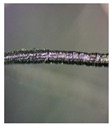
NaOH	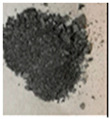	n.a.	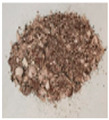	n.a.	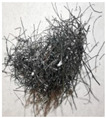	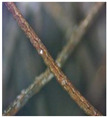
Comments (Test temperature)	Failed (T = 450 °C)		Failed (T = 450 °C)		Passed (T = 340 °C)	

## Data Availability

Data available in https://doi.org/10.34810/data1218.
